# DNA topoisomerase IIβ stimulates neurite outgrowth in neural differentiated human mesenchymal stem cells through regulation of Rho-GTPases (RhoA/Rock2 pathway) and Nurr1 expression

**DOI:** 10.1186/s13287-018-0859-4

**Published:** 2018-04-25

**Authors:** Merve Zaim, Sevim Isik

**Affiliations:** SANKARA Brain and Biotechnology Research Center, Entertech Technocity, Avcılar, 34320 Istanbul, Turkey

**Keywords:** Human mesenchymal stem cells, Neural differentiation, Neurite outgrowth, DNA topoisomerase IIβ, Rho GTPases, Neurodegeneration

## Abstract

**Background:**

DNA topoisomerase IIβ (topo IIβ) is known to regulate neural differentiation by inducing the neuronal genes responsible for critical neural differentiation events such as neurite outgrowth and axon guidance. However, the pathways of axon growth controlled by topo IIβ have not been clarified yet. Microarray results of our previous study have shown that topo IIβ silencing in neural differentiated primary human mesenchymal stem cells (hMSCs) significantly alters the expression pattern of genes involved in neural polarity, axonal growth, and guidance, including Rho-GTPases. This study aims to further analyze the regulatory role of topo IIβ on the process of axon growth via regulation of Rho-GTPases.

**Methods and results:**

For this purpose, topo IIβ was silenced in neurally differentiated hMSCs. Cells lost their morphology because of topo IIβ deficiency, becoming enlarged and flattened. Additionally, a reduction in both neural differentiation efficiency and neurite length, upregulation in RhoA and Rock2, downregulation in Cdc42 gene expression were detected. On the other hand, cells were transfected with topo IIβ gene to elucidate the possible neuroprotective effect of topo IIβ overexpression on neural-induced hMSCs. Topo IIβ overexpression prompted all the cells to exhibit neural cell morphology as characterized by longer neurites. RhoA and Rock2 expressions were downregulated, whereas Cdc42 expression was upregulated. Nurr1 expression level correlated with topo IIβ in both topo IIβ-overexpressed and -silenced cells. Furthermore, differential translocation of Rho-GTPases was detected by immunostaining in response to topo IIβ.

**Conclusion:**

Our results suggest that topo IIβ deficiency could give rise to neurodegeneration through dysregulation of Rho-GTPases. However, further in-vivo research is needed to demonstrate if re-regulation of Rho GTPases by topo IIβ overexpression could be a neuroprotective treatment in the case of neurodegenerative diseases.

**Electronic supplementary material:**

The online version of this article (10.1186/s13287-018-0859-4) contains supplementary material, which is available to authorized users.

## Background

Type II DNA topoisomerases (topo II) are involved in double-strand cleavage and rejoining of nuclear DNA. In mammalian cells, there are two isoforms of topo II: topo IIα and topo IIβ [1, 2]. Unlike topo IIα, which is closely correlated with chromosomal segregation in dividing and pluripotent cells, topo IIβ is involved in more specific processes and is intensively expressed in postmitotic and differentiated tissues [3, 4].

Studies have demonstrated that topo IIβ has a significant role in neural differentiation during brain development by potentiating inducible neuronal genes to become transcribable [5–9]. Topo IIβ inhibition in cultured cerebellar granule neurons (CGNs), dorsal root ganglions (DRGs), cortical neurons (CNs), and PC12 cells undergoing nerve growth factor (NGF)-induced differentiation significantly blocked neurite outgrowth and growth cone formation. Failure of neurons from topo IIβ knockout mice to contact with muscle cells in cocultures could be due to the lack of topo IIβ-mediated neurite outgrowth [10]. In agreement with previous studies, we showed a decrease in neurite length due to topo IIβ silencing of neural differentiated human mesenchymal stem cells (hMSCs) [11]. Topo IIβ may be involved in the regulation of certain gene expressions required for neurite outgrowth during neural differentiation [10, 12, 13].

Neurite outgrowth and growth cone formation are substantial steps in neuronal development [14]. Rho-GTPase family members (RhoA, Cdc42, Rac1), which are major intracellular regulators of neuronal polarity, integrate signals generated from extracellular matrix and reorganize the actin cytoskeleton, thereby arranging the morphology of neurites and growth cones [10, 15].

RhoA, a negative regulator of neurite outgrowth, induces growth cone collapse and neurite retraction. Rock2 is the major downstream target of RhoA in neural cells, and activation of RhoA results in axon growth inhibition through the RhoA/Rock2 pathway [16, 17]. Unlike RhoA, Rac1 and Cdc42 give rise to an increase in growth cone development and neurite outgrowth [18, 19]. Moreover, these small GTPases regulate neuronal survival and death. Similar to their opposing functions in neurite outgrowth and growth cone formation, Cdc42/Rac1 activation promotes neuronal survival while Rho activation often elicits neuronal death [18, 19]. Furthermore, Rho-GTPases play crucial roles in mediating nervous system development and neuronal survival and, thus, dysregulation of these GTPases lies behind the pathology of several neurodegenerative diseases including Alzheimer’s disease (AD), Parkinson’s disease (PD), Huntington’s disease (HD), and amyotrophic lateral sclerosis (ALS) [17, 19].

In a previous study of ours, we investigated the target genes regulated by topo IIβ in neural differentiation of hMSCs. According to the microarray results, topo IIβ silencing appears to be important in several signal transduction pathways that contribute to neuronal polarization and pathogenesis of neurodegenerative diseases. In particular, genes involved in the regulation of neuronal cell morphology and cytoskeletal organization (e.g., Cofilin1, SORBS2) were found to be regulated by topo IIβ [11]. Microarray analysis revealed that RND1 and RND2, atypical Rho members that are considered constitutively active and that antagonize the action of RhoA in the cytoskeletal organization by inducing neurite extensions, were downregulated in response to topo IIβ silencing [11]. For further analysis, this study aims to evaluate the possible regulatory role of topo IIβ on Rho-GTPases in axonal growth of neural differentiated hMSCs.

Neural differentiated MSCs are excellent tools and models for the treatment of nervous system disorders [20–23]. Although previous studies have shown that topo IIβ promotes axonal growth in primary neurons [10], topo IIβ-related studies performed on hMSC-derived neural cells are very limited. Parenthetically, there have been no detailed report elucidating the downstream pathway of topo IIβ in the regulation of neurite outgrowth.

In the present study, we discriminated the genes regulated by topo IIβ in differentiation and neurite outgrowth by differentiating hMSCs into neural cells. Initially, we silenced topo IIβ expression with specific small interfering (si)RNAs and examined how morphology, neural differentiation potential, axon growth, and Rho-GTPase expressions are affected by topo IIβ deficiency. Then, we investigated the possible neuroprotective effect of topo IIβ overexpression on neural differentiated hMSCs and elucidated the further involvement of Rho-GTPases in topo IIβ-mediated axon growth.

## Methods

In this study, the hMSC line was assembled into six individual groups according to neural differentiation, topo IIβ silencing, and overexpression (Table 1).

**Table 1:** Experimental groups and abbreviations used in the study

Abbreviations	Experimental groups
hMSC	Control (untreated) human mesenchymal stem cells (hMSCs)
hMSC_mN3	Neural differentiated hMSCs
hMSC_topo IIβ(−)	Topo IIβ transfected (silenced) hMSCs with siRNAs
hMSC_topo IIβ(+)	Topo IIβ transfected (overexpressing) hMSCs with pEGFP_topo IIβ
hMSC_topo IIβ(−)_mN3	Topo IIβ transfected (silenced) hMSCs with siRNAs induced to neural differentiation
hMSC_topo IIβ(+)_mN3	Topo IIβ transfected (overexpressing) hMSCs with pEGFP_topo IIβ induced to neural differentiation

### Culture of bone marrow-derived hMSC line

The bone marrow-derived hMSC (BM-hMSC) line (UE7T-13 cells, no. RBRC-RCB2161), infected with retroviruses expressing papillomavirus E7 and hTERT to extend the lifespan of the cells [24–28], was purchased from Riken Science Institute, Japan. Cells were detached using 0.25% Trypsin/EDTA (Gibco) solution when culture reached 80–90% confluency. The BM-hMSC line was seeded at a density of 3 × 10^3^ cells/cm^2^ in expansion medium (Dulbecco’s modified Eagle’s medium low glucose (DMEM-LG), 10% fetal bovine serum (FBS), 0.1 mg/ml primocin) and incubated at 37 °C, in a 5% CO_2_ incubator. Subculture was repeated every 4 days.

### Immunophenotyping of hMSCs

The hMSC line was analyzed by flow cytometry for the expression of hMSC specific cell surface antigens. Commonly used antibodies—CD45 (FITC), HLA-DR (PerCP), CD34 (PE), CD73 (APC), CD90 (FITC), and CD105 (PE)—were used to characterize hMSC populations.

### Mesodermal differentiation of hMSCs

#### Adipogenic differentiation

For adipogenic differentiation, the hMSC line was harvested at passage 3 (p3) and seeded into 24-well plates at a density of 5 × 10^3^ cells/cm^2^. Cells were treated with complete MesenCult adipogenic medium containing MesenCult MSC basal medium (Stemcell) and 10% adipogenic stimulatory supplement (Stemcell) for 21 days; adipogenic differentiation was confirmed by Oil Red O staining.

#### Chondrogenic differentiation

For chondrogenic differentiation, the hMSC line was harvested at p3 and seeded into 24-well plates at a density of 7.5 × 10^5^ cells/cm^2^. To stimulate chondrogenic differentiation, the culture medium was replaced with Stempro chondrocyte differentiation basal medium (Gibco) containing 10% Stempro chondrogenesis supplement (Gibco). After 21 days of cultivation, the chondrogenic pellet was stained with Alcian Blue.

#### Osteogenic differentiation

For osteogenic differentiation, the hMSC line was seeded into 24-well plates at a density of 2 × 10^5^ cells/cm^2^. Osteogenic differentiation was stimulated by refreshing the expansion medium with complete MesenCult osteogenic medium including MesenCult MSC basal medium, osteogenic stimulatory supplement, dexamethasone, and ascorbic acid (all from Stemcell). After 5 days, when multilayering had been observed, β-glycerophosphate was added to complete MesenCult osteogenic medium. The chondrogenic pellet was assessed with Toluidine Blue staining after 5 weeks of cultivation.

### Neural differentiation of hMSCs

hMSCs were seeded in culture medium at a density of 5 × 10^3^ cells/cm^2^ prior to neural induction. The induction medium was composed of several cytokines and growth factors, including 0.5 mg/ml dibutyryl cyclic AMP (dbcAMP; Sigma), 0.5 mM 3-isobutyl-1-methylxanthine (IBMX; Sigma), 20 ng/ml human epidermal growth factor (hEGF; Sigma), 40 ng/ml recombinant human fibroblast growth factor (rhFGF; R&D systems), 10 ng/ml fibroblast growth factor (FGF-8; Pepro Tech), 10 ng/ml recombinant human brain-derived neurotrophic factor (rhBDNF; R&D systems), 2 mM l-glutamine (Gibco), and 40 ng/ml NGF in neurobasal medium (Gibco) supplemented with 2% B27 supplement (Gibco), and defined as modified N3 medium (mN3) as previously described [11]. Culture medium was changed with mN3 medium to induce neural differentiation. Cells in hMSC_topo IIβ(−)_mN3 and hMSC_topo IIβ(+)_mN3 groups were induced to neural differentiation 48 h post-transfection. mN3 medium was refreshed every 48 h for 5 days. Morphology of the cells was observed under an inverted phase contrast microscope.

### Immunostaining

The hMSC line was permeabilized with TZN buffer (10 mM pH 7.5 Tris–HCl, 0.5% Nonidet P-40, 0.2 mM ZnCl_2_) and fixed with 4% paraformaldehyde (PFA)/phosphate-buffered saline (PBS). After blocking with 10% normal goat serum (NGS; Gibco) and 10% normal horse serum (NHS; Biochrom) in 0.3% PBS/Triton X (PBS-Tx), cells were treated with specific primary antibodies in PBS-Tx with 3% NHS. Antibodies against neurofilament (NF; 1:100, Millipore), microtubule-associated protein (MAP2; 1:100, Promega), Tau (1:100, Santa Cruz), RhoA (1:100, Santa Cruz), Rac1 (1:100, BD), and Cdc42 (1:100, Santa Cruz) were used at the indicated dilutions. Following the washing steps, cells were treated with the secondary antibodies GAM-IgG-Alexa Fluor 488 (1:100, Invitrogen) and GAR-IgG-Alexa Fluor 594 (1:100, Invitrogen). Cells were then treated with 1:15,000× DAPI (Sigma) and, after final washings with PBS and distilled water, the slides were observed under a fluorescent microscope (Carl Zeiss).

### Silencing of topo IIβ in hMSCs by siRNAs

#### Determination of silencing efficiency

Lipofectamine RNAiMAX reagent and four different validated topo IIβ-specific siRNAs were used to silence topo IIβ expression at the mRNA level. The hMSC line was seeded at a density of 6 × 10^3^ cells/cm^2^. Briefly, siRNAs (7.5, 10, 12.5, 15, and 20 nM) and reagent were diluted with Optimem, and siRNA-Lipofectamine RNAiMAX complexes were added to the cells. At 48 h post-transfection, total RNA of the topo IIβ-silenced hMSC line was extracted and efficiency was checked by real-time quantitative polymerase chain reaction (RT-qPCR). siRNA transfection was repeated every 48 h for 5 days for the hMSC_topo IIβ(−) and hMSC_topo IIβ(−)_mN3 groups.

### Cell viability

To determine the optimum concentration of topo IIβ-specific siRNAs on the hMSC line, an MTT cell proliferation assay (Roche) was performed. The hMSC line was seeded in 96-well plates at a density of 2 × 10^3^ cells /well. Four different validated siRNAs (topo IIβ-5, topo IIβ-6, topo IIβ-7, and topo IIβ-8; Qiagen) and Lipofectamine RNAiMAX reagent were used for silencing topo IIβ. siRNAs (7.5, 10, 12.5, 15, and 20 nM) and reagent were diluted with Optimem and incubated for 15 min at room temperature to allow siRNA-Lipofectamine RNAiMAX complex formation. siRNA-Lipofectamine RNAiMAX complexes were added to the wells. After 48 h of siRNA transfection, a cell proliferation assay was performed with the MTT reagent (Roche) according to the manufacturer’s instructions. Absorbance was measured at 490 nm using a microplate reader (Synergy HT; Biotek).

### Transfection of hMSCs with topo IIβ gene

#### Topo IIβ plasmid isolation and purification

The pEGFP-N1 plasmid including topo IIβ gene was kindly gifted by Prof. Tsutsui from Okayama University Medical School. The EGFP-N1 plasmid was transformed to calcium shocked competent *E. coli* DH5α strain for amplification and plasmid isolation was performed with the Plasmid Isolation Kit (Qiagen) according to the manufacturer’s instructions. Before transfection, confirmation of topo IIβ gene insert was carried out by digestion of the plasmid with appropriate restriction enzymes (XhoI, SmaI, and BamHI) and colony PCR.

### Determination of overexpression efficiency

To transfect the hMSC line with topo IIβ gene, 4D Nucleofector™ system (Lonza) was used; 5 × 10^5^ cells of the hMSC line were transfected with five different concentrations of topo IIβ plasmid (4, 5, 6, 8, and 10 μg) using the 4D Nucleofector™ system (Lonza). hMSCs were detached by 0.25% Trypsin/EDTA (Gibco) and resuspended in a total of 100 μl of solution, including 82 μl nucleofector solution and 18 μl supplement. Topo IIβ plasmid (4, 5, 6, 8, and 10 μg) were added to each 100 μl solution and transferred into nucleocuvettes, respectively. The FF-104 (high-efficiency) program was applied. After nucleofection, cells were resuspended in 500 μl prewarmed RPMI containing 10% FBS and incubated at 37 °C for 10 min as a recovery step. The transfected hMSC line was seeded into culture dishes containing the DMEM-LG and 10% FBS and incubated at 37 °C in a 5% CO_2_ incubator. The medium was refreshed 24 h after nucleofection. Total RNA was extracted and the efficiency of overexpression was measured by RT-qPCR (Corbett Life Science).

### Cell viability

To determine the plasmid concentration-based cytotoxicity, an MTT viability assay was performed. Briefly, the hMSC line was detached and resuspended in a total of 100 μl of nucleofection solution including 4, 5, 6, 8, and 10 μg of topo IIβ plasmid and transferred into nucleocuvettes, respectively. After nucleofection and a recovery step, cells were seeded into culture dishes containing the DMEM-LG and 10% FBS and incubated at 37 °C in a 5% CO_2_ incubator. After 48 h of topo IIβ transfection, a cell proliferation assay was performed with the MTT reagent (Roche) according to the manufacturer’s instructions. Absorbance was measured at 490 nm using a microplate reader (Synergy HT; Biotek).

### Reverse transcription quantitative PCR (RT-qPCR)

Total RNA was extracted using the RNeasy kit (Qiagen) and reverse transcribed into cDNA by the Quantitect Reverse Transcription Kit (Qiagen) according to the manufacturer’s instructions.

Real-time PCR amplification was carried out using cDNA samples, gene specific primers, ddH_2_O, and the SYBR Premix Ex Taq (Tli RNase H Plus) including second generation dye SYBRGreen on a Rotor Gene 6000 Real Time PCR instrument (Corbett Life Science) under given thermocycling conditions. Expression levels of GAPDH, Topo IIβ, RhoA, Cdc42, Rac1, Rock2, and Nurr1 primers were determined using the ∆∆Ct formula according to Pfaffl [29]. Primer sequences are indicated in Table 2. A standard dilution graph was drawn according to the GAPDH primer. Ct values of the remaining primers were normalized according to a standard dilution graph. Triplicate samples were used, and experiments were repeated three times.

**Table 2:** Primer sequences for reverse transcription quantitative polymerase chain reaction (RT-qPCR)

Primers	Forward	Reverse	Annealing Temperature
GAPDH	GCGAGATCCCTCCAAAATCAA	GTTCACACCCATGACGAACAT	60 °C
Topo IIβ	TTTTTCACCATCATTTGGTCTG	GGGCTTAGGGACTGTATCTGAA	60 °C
RhoA	CTGGTGATTGTTGGTGATGG	GCGATCATAATCTTCCTGCC	55 °C
Rac1	AACCAATGCATTTCCTGGAG	CAGATTCACCGGTTTTCCAT	60 °C
Cdc42	CTCCGGAAACTCAACCCAAA	GACGCAGAGGCTTTCAAACAG	60 °C
Rock2	TTGCTCTGGATGCAATACACTC	TCTCGCCCATAGAAACCATCA	55.5 °C
Nurr1	CTTGTGTTCAGGCGCAGTATG	GAGTGGTAACTGTAGCTCTGAGAAGC	60 °C

### Neurite outgrowth

Neurite length per neural cell was determined as the sum of the lengths of all neurites of a single neural cell, measured with NIS Elements software (Nikon) and calculated using the ImageJ software (NIH). The average total neurite length per group was determined from at least 100 images from random fields. Images were investigated not for only length measurements but also calculation of neural differentiation efficiency.

### Statistical analyses

Data are presented as mean ± standard deviation (SD) of three independent experiments. The paired *t* test was used to evaluate the differences between the experimental groups. Differences were considered statistically significant at *p < 0.01 and **p < 0.001.

## Results

### mN3 cytokine combination induced successful neural differentiation in hMSCs

To evaluate the neural differentiation of the BM-hMSC line, we initially demonstrated that our data were in agreement with the criteria of the International Society for Cellular Therapy. BM-hMSCs were adherent to plastic in culture with typical fibroblastic morphology, and cells maintained their morphology up to p30 under culture conditions.

For characterization of the hMSC line, the immunophenotypic cell surface profile for CD45, HLA-DR, CD105, CD34, CD73, and CD90 were analyzed by flow cytometry; cells were positive for the markers CD73, CD90, and CD105, and negative for CD45, CD34, and HLA-DR (Additional file 1: Figure S1).

The multilineage mesodermal differentiation potential of the hMSC line was assessed by differentiating cells into adipocytes, osteocytes, and chondrocytes by incubating under appropriate in-vitro conditions. Lipid vacuoles in adipocytes were observed 3 weeks after adipogenic induction, following staining with Oil Red O. After 5 weeks, hMSCs generated aggregates or nodules that were stained positive by Toluidine Blue under osteogenic culture conditions, and calcium accumulation was obtained. Chondrogenic cell pellets showed strong histological staining with Alcian Blue for the presence of proteoglycans (Additional file 2: Figure S2).

**Fig. 1:**
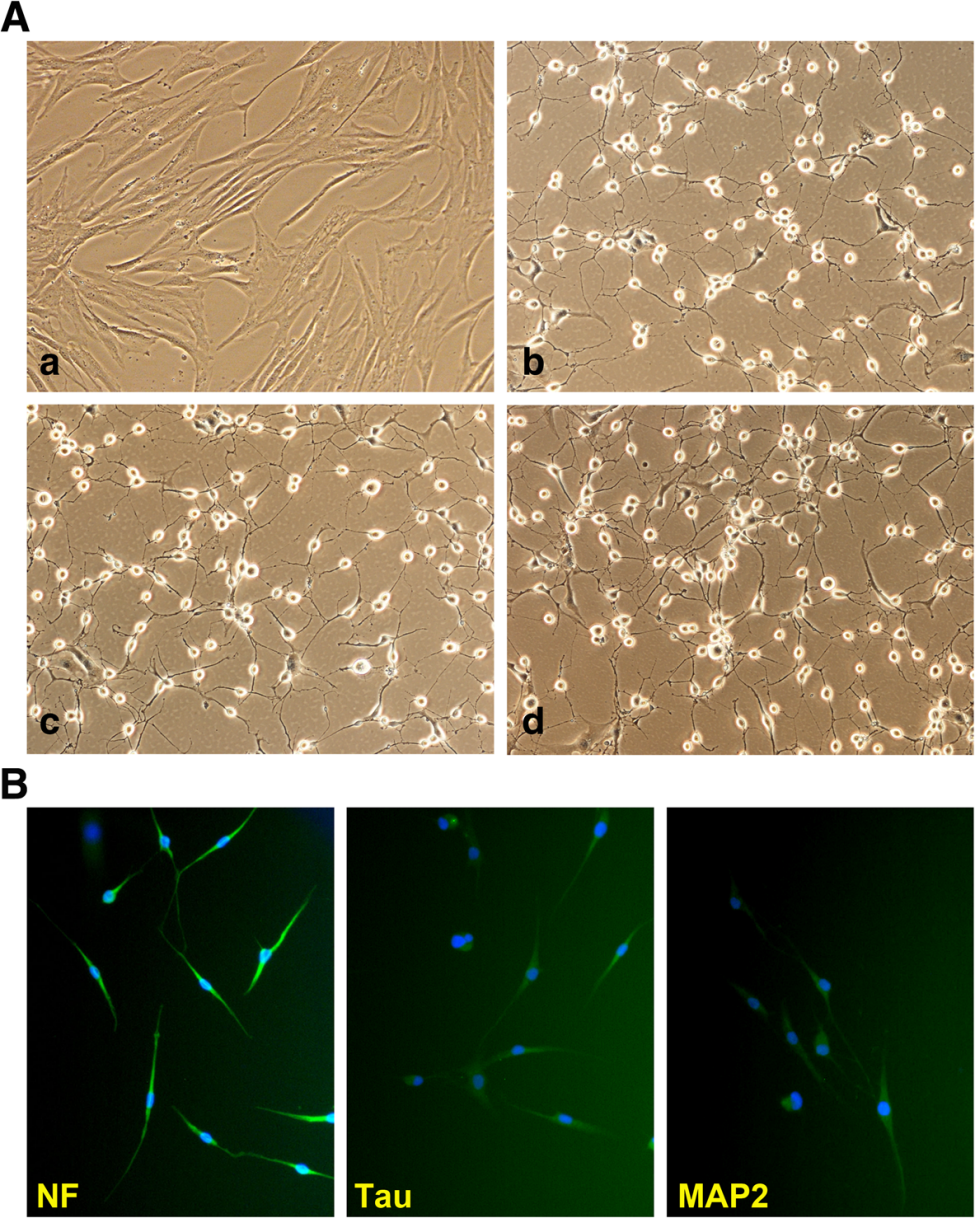
Neural differentiation of hMSCs with the mN3 cytokine combination. **A** Undifferentiated hMSCs (control, **a**), neural differentiated hMSCs at day 1 (**b**), day 3 (**c**) and day 5 (**d**). mN3 treatment induced hMSC cellular differentiation characterized by a polar appearance with long, out-branching axons. **B** Immunostaining of neural differentiated hMSCs at day 5 with the neural markers neurofilament (NF), Tau, and microtubule-associated protein (MAP2). mN3 treatment induced the expression of neural differentiation markers (images ×10)

The hMSC line was induced to neural differentiation by treating cells with the mN3 cytokine combination for 5 days in culture. mN3, a variation of N3 neural induction cocktail first suggested by Long et al. [30], is a nontoxic cytokine combination with high neural differentiation efficiency. Throughout the differentiation process, cells were seen to structurally adopt a polarized phenotype with longer neurites generating a neuronal network within 24 h while they were still in the process of neural maturation (Fig. 1A). To confirm neural differentiation of the hMSC line, immunofluorescent staining was performed 5 days after induction. Neural differentiated hMSCs stained positive for the neural markers NF (98%), Tau (99%), and MAP2 (100%) (Fig. 1B). No glial marker expression (glial fibrillary acidic protein (GFAP)) was seen. Taken together, the results demonstrate that mN3 induced cell morphology changes consistent with neural differentiation, and this was validated by increased expression of known neural differentiation markers. These findings demonstrate that mN3 treatment enhances differentiation of the hMSC line in vitro.

### Topo IIβ expression is crucial for neural differentiation and neurite outgrowth of neural-induced hMSCs

**Fig. 2:**
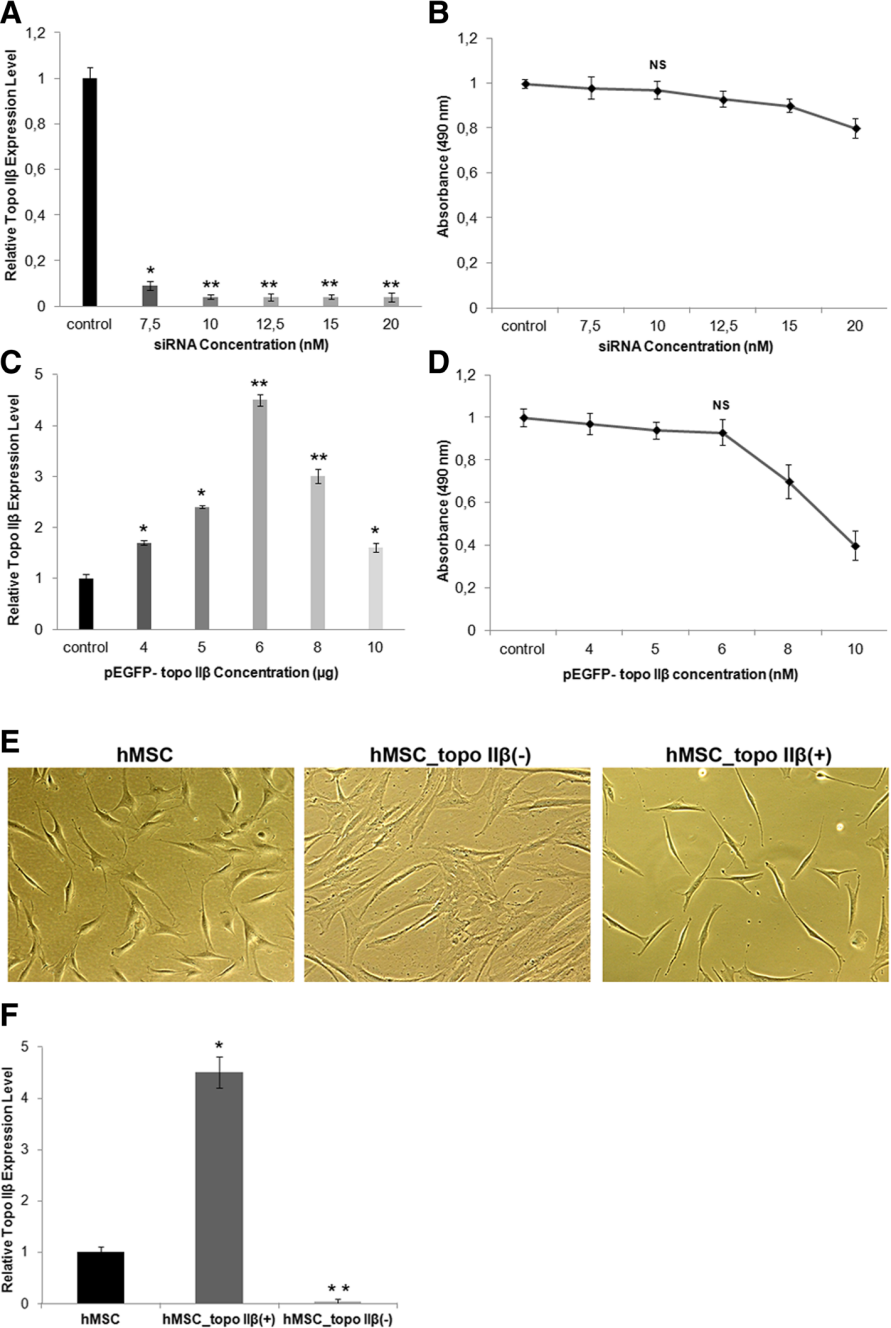
Detection and quantification of topoisomerase (topo) IIβ overexpression and silencing in hMSCs. **a** Topo IIβ was silenced using 7.5, 10, 12.5, 15, and 20 nM topo IIβ-specific small interfering (si)RNAs and silencing efficiency was determined by RT-qPCR. **b** siRNA-based cytotoxicity for each siRNA concentration was evaluated by MTT cell proliferation assay. **c** Topo IIβ was overexpressed using 4, 5, 6, 8, and 10 μg pEGFP-topo IIβ and transfection efficiency was quantified by RT-qPCR. **d** EGFP-topo IIβ plasmid-based cytotoxicity for each plasmid concentration was evaluated by MTT cell proliferation assay. **e** Morphology of control (untransfected hMSCs), topo IIβ-silenced, and -overexpressed hMSCs. **f** Topo IIβ expression levels of topo IIβ-silenced (10 nm siRNA) and -overexpressed (6 μg pEGFP-topo IIβ) hMSCs were compared by RT-qPCR. Images ×10. Error bars represent the means ± standard deviation; *p < 0.01, **p < 0.001. NS, nonsignificant

Topo IIβ expression was silenced using four different validated siRNAs targeting human topo IIβ mRNA. To detect the optimum (> 90%) topo IIβ-specific siRNA concentration, 7.5, 10, 12.5, 15, and 20 nM siRNAs was used. RT-qPCR results of topo IIβ-silenced hMSCs showed that 7.5 nM siRNA can silence topo IIβ gene expression (85 ± 2%) (*p* < 0.01); however, maximal knockdown (> 95%) (*p* < 0.001) was obtained using 10 nM siRNA, and increasing the siRNA concentration from 10 nM to 20 nM did not enhance the silencing efficiency (Fig. 2a).

A cell proliferation and cytotoxicity assay based on MTT was performed to elucidate the effects of different concentrations of siRNAs on proliferation of the hMSC line. A cell viability graph of untransfected and topo IIβ-silenced hMSCs revealed that viability of transfected cells was not affected by siRNAs compared with untransfected cells. Results indicated that siRNA concentrations from 7.5 to 20 nM did not have a significant cytotoxic effect on cells (Fig. 2b). We obtained highly efficient siRNA transfection (> 95%) using 10 nM siRNA without any significant cytotoxicity (Fig. 2b).

The plasmid pEGFP-topo IIβ, which encodes the full-length rat topo IIβ with enhanced green fluorescent protein (EGFP) sequence fused at the C-terminus, was constructed and kindly gifted by Prof. Tsutsui from Okayama University Medical School, Japan. Transfection of the hMSC line with different concentrations of topo IIβ plasmid (4, 5, 6, 8, and 10 μg) was performed using the FF-104 (high-efficiency) program of the 4D Nucleofector™ system (Lonza) to determine the optimum plasmid concentration with maximum transfection efficiency and minimum cytotoxicity. Transfection of hMSCs with 6 and 8 μg plasmid resulted in the highest topo IIβ gene expression level among other transfected cells (Fig. 2c).

The MTT cell proliferation assay was performed to determine specific toxicity of each topo IIβ plasmid concentration (4, 5, 6, 8, and 10 μg) on the hMSC line. According to the graph (Fig. 2d), increasing the plasmid concentration up to 6 μg did not have any cytotoxic effect on transfected cells compared with control cells. Therefore, 6 μg plasmid was used in further transfection experiments.

To identify whether topo IIβ silencing or overexpression alters the cell morphology, hMSCs were transfected with siRNAs or pEGFP_topo IIβ without neural induction. The untransfected (control) hMSC line had fibroblastic and spindle-shaped morphology, whereas topo IIβ-silenced hMSCs lost their characteristic morphology and became enlarged and flattened. Only GFP-transfected cells showed the same morphology as untransfected cells (data not shown). Topo IIβ transfected hMSCs were fibroblastic and spindle-shaped as well and maintained their morphology after transfection, furthermore gaining longer and thinner cellular processes. Morphological changes were observed 24 h after transfection (Fig. 2e). RT-qPCR was performed to confirm overexpression and silencing of topo IIβ in the hMSC line at the mRNA level. Figure 2f shows a 4.5-fold increase (*p* < 0.01) in hMSC_topo IIβ(+) and a 25-fold (*p* < 0.001) suppression in hMSC_topo IIβ(-) compared with untransfected cells. This result indicates a sufficient overexpression and knockdown of cells 48 h post-transfection.

**Fig. 3:**
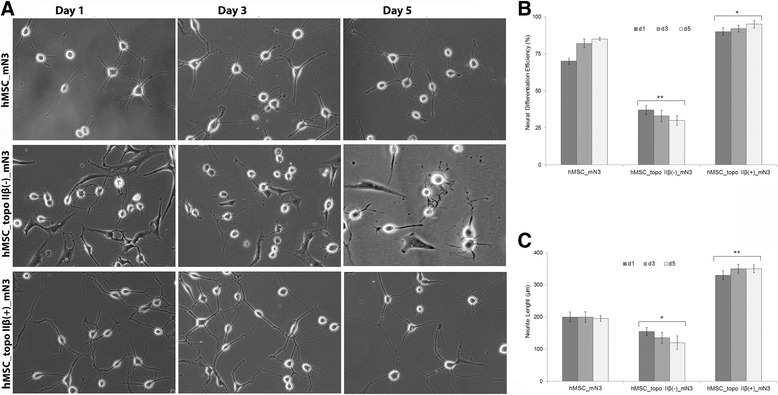
Neural differentiation of untransfected, topo IIβ-silenced, and -overexpressed hMSCs. **a** Morphology of untransfected, topo IIβ-silenced, and -overexpressed hMSCs induced to neural differentiation by the mN3 cytokine combination for 5 days. **b** Neural differentiation efficiencies and **c** neurite lengths of topo IIβ-silenced and -overexpressed cells at day (d)1, day 3, and day 5 were compared with untransfected cells. Both neural differentiation efficiency and neurite length were directly proportional to topo IIβ expression. The average total neurite length and neural differentiation efficiency per group for each day was determined from at least 100 images from random fields. Images ×10. Error bars represent the means ± standard deviation; *p < 0.01, **p < 0.001

To identify how topo IIβ silencing or overexpression affects the neural differentiation potential and neurite lengths of neural-induced hMSCs, we assessed the differentiation potential and neurite lengths of each group over 5 days. Neural differentiation efficiencies and neurite lengths of untransfected, topo IIβ-silenced, and overexpressing hMSC line were compared at days 1, 3, and 5 (Fig. 3). Neural differentiation efficiency was quantified by determining the percentage of cells both positive for neural markers and having neural-like morphology. As shown in Fig. 3b, untransfected cells exhibited high levels of neural differentiation potential starting from 70 ± 2% at day 1 and reached 85 ± 5 at day 5. The neurogenic potential of topo IIβ-deficient cells was extremely low (37 ± 3% at day 1) compared with untransfected cells (*p* < 0.001) and decreased during the differentiation process (30 ± 4% at day 5). On the other hand, transfection of the hMSC line with topo IIβ resulted in an increased neural differentiation efficiency from 90 ± 3% (day 1) to 95 ± 5% (day 5) (*p* < 0.01). Neurite lengths were measured using the NIS Elements software (Nikon) and ImageJ (NIH) program as described in the Methods section. Neurite outgrowth in topo IIβ-deficient cells decreased from 200 ± 15% to 155 ± 12% at day 1, and 195 ± 9% to 120 ± 18% at day 5 compared with untransfected cells (*p* < 0.01). Measurements of the neurite lengths of topo IIβ-overexpressing cells showed increased neurite length, with 330 ± 12% at day 1 and 350 ± 8% at day 5 (*p* < 0.001) (Fig. 3c). Results indicate that topo IIβ silencing decreased the neural differentiation potential of the hMSC line as most cells were unresponsive to differentiation and inhibited neurite outgrowth, whereas topo IIβ overexpression increased the neural differentiation potential of mN3-induced hMSCs since almost all cells showed neuronal morphology and induced neurite outgrowth (Fig. 3).

### Topo IIβ regulates axon growth and maintenance through Rho-GTPases in neural differentiated hMSCs

**Fig. 4:**
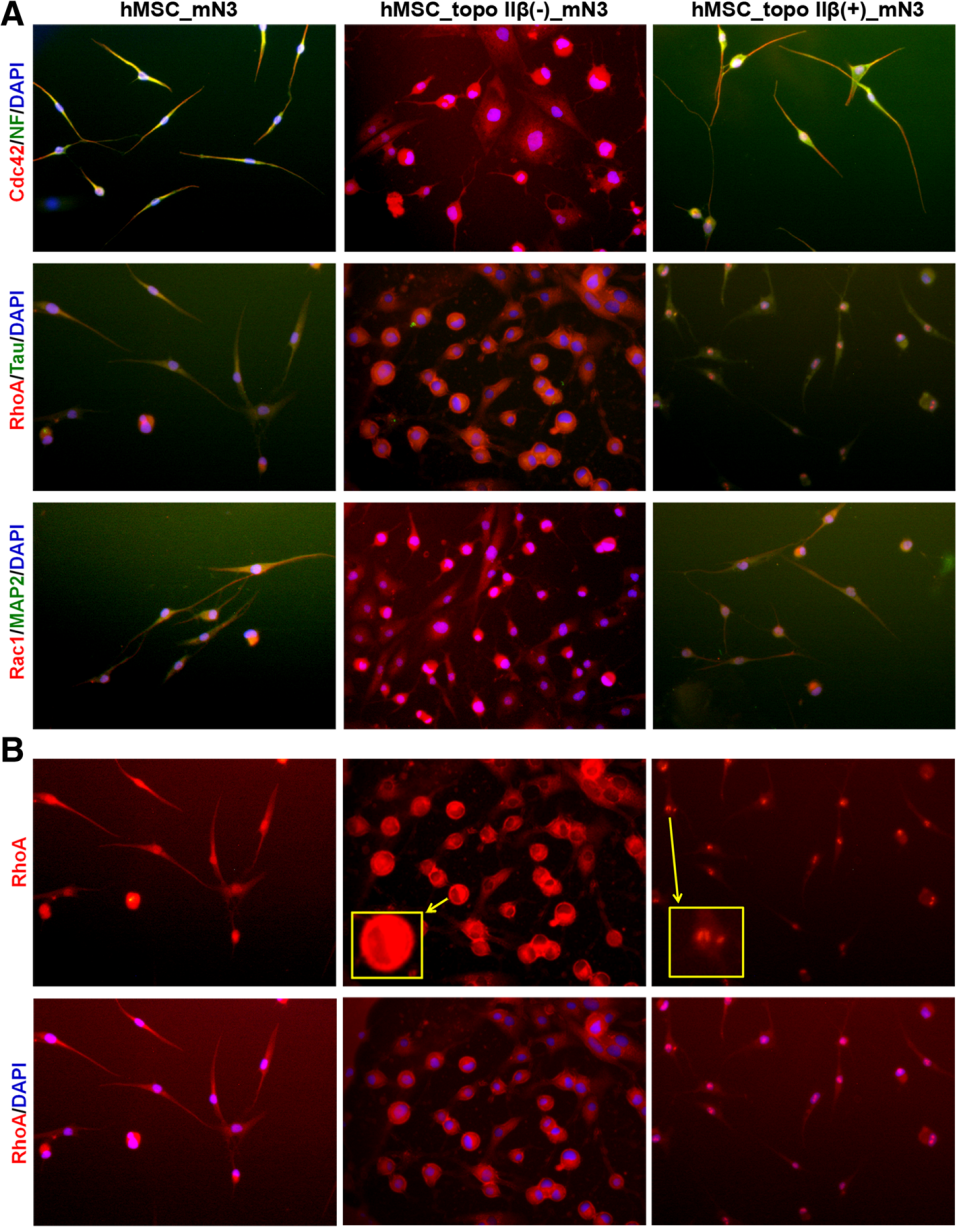
Immunostaining of hMSC_mN3, hMSC_topo IIβ(−)_mN3, and hMSC_topo IIβ(+)_mN3 cells with the neural markers neurofilament (NF), Tau, microtubule-associated protein (MAP2), and Rho-GTPases Cdc42, RhoA, and Rac1. In hMSC_mN3 cells, the arrows indicate the overlapping of the neural marker NF and Rho-GTPase Cdc42 expressions (yellow). In hMSC_topo IIβ(+)_mN3 cells, the arrows indicate Rho-GTPase Cdc42 expression in extending the ends of axons (red) (**a**). Immunostaining of hMSC_mN3, hMSC_topo IIβ(−)_mN3 and hMSC_topo IIβ(+)_mN3 cells with RhoA. Arrows indicate the lack of RhoA expression in the nucleus of hMSC_topo IIβ(−)_mN3 and the enhanced RhoA expression in the nucleus of hMSC_topo IIβ(+)_mN3 cells (**b**). Images ×10, magnified nucleus ×20

Untransfected, topo IIβ-silenced, and overexpressed hMSCs were induced to neural differentiation with the mN3 cytokine combination, and cells were immunostained with Rho-GTPase (RhoA, Cdc42, Rac1) and neural marker (Tau, NF, MAP2) antibodies on the fifth day of neural induction (Fig. 4).

Immunofluorescence staining showed that all the neural differentiated hMSCs stained positively for the neural markers NF, Tau, and MAP2. In neural differentiated cells lacking topo IIβ expression, the neural differentiation efficiency decreased, and nondifferentiated cells expanded morphologically and their nuclei grew in size. As a result of the diminished neural differentiation efficiency, the expression of the neural markers was reduced (the dominant color was red).

Rho-GTPase expressions were detected in both the cytoplasm and the nucleus, but their localizations were changed among groups. While colocalization of the neural marker and Rho-GTPase expressions were detected in untransfected neural cells (shown in yellow in Fig. 4a), Rac1 and especially Cdc42 immunoreactivity (shown in red in Fig. 4a) was enriched in the growing tips of prolonged axons in topo IIβ-overexpressing cells (Fig. 4a).

RhoA was found to be expressed in both the cytoplasm and the nucleus of untransfected cells. However, RhoA expression was not detected in the nucleus of topo IIβ-silenced cells. On the other hand, unlike topo IIβ-deficient cells, RhoA expression was enriched in the nuclear regions and was especially localized in the nucleolus in topo IIβ-overexpressing cells (Fig. 4b).

### Topo IIβ regulates expression of Nurr1, Rho-GTPases (Cdc42, RhoA, Rac1), and Rock2

**Fig. 5:**
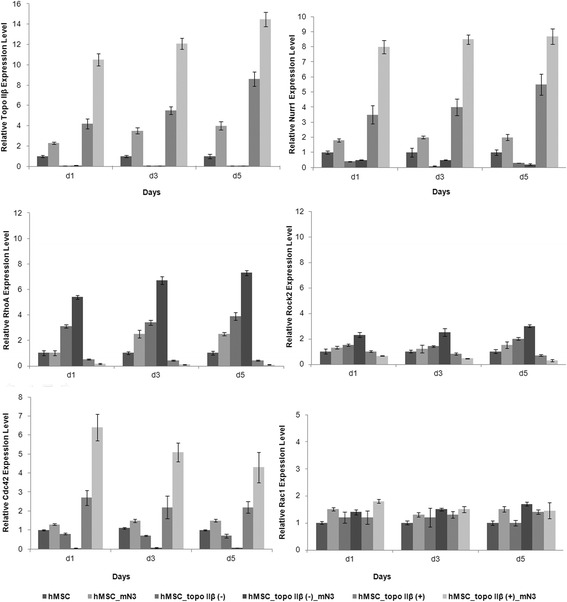
RT-qPCR results of relative Topo IIβ, Nurr1, Rho GTPase (RhoA, Rac1, and Cdc42), and Rock2 expressions at days (d)1, 3, and 5 for each group. Nurr1 showed nearly the same pattern as topo IIβ, since the expression increased due to neural differentiation and topo IIβ transfection. Cdc42 also exhibited a similar expression pattern to topo IIβ and Nurr1, being upregulated due to neural induction and topo IIβ overexpression. RhoA and the downstream effector kinase Rock2 expressions were inversely correlated with topo IIβ, Nurr1, and Cdc42 expressions. Neural induction and topo IIβ overexpression increased Nurr1 and Cdc42 expression, whereas decreased RhoA and Rock2. Rac1 expression was independent. Error bars represent the means ± standard deviation; *n* = 3 samples in triplicate; *p < 0.01, **p < 0.001

Relative quantification was performed to calculate the RT-qPCR results of Topo IIβ, Nurr1, Cdc42, RhoA, Rac1 and Rock2 gene expressions (Fig. 5). A standard curve using the housekeeping gene GAPDH was used as an internal control and, according to the curve, RT-qPCR efficiency was over 99% which is within the accepted range of efficiency.

Cells in each experimental group were cultured for 5 days and mRNA levels of topo IIβ gene in each group were determined at days 1, 3, and 5 by RT-qPCR. The topo IIβ expression pattern of hMSC_mN3 cells gradually increased with the number of days in culture compared with untransfected hMSCs. As a result of topo IIβ knockdown, expression levels were suppressed in hMSC_topo IIβ(−) and hMSC_topo IIβ(−)_mN3 cells (*p* < 0.001). hMSC_topo IIβ(+) cells exhibited higher topo IIβ expression levels compared with untransfected cells (*p* < 0.001). The highest topo IIβ levels were detected in hMSC_topo IIβ(+)_mN3 cells and the expression pattern increased with days in culture (*p* < 0.001).

The expression level of RhoA, a well-known negative regulator of neural differentiation and neurite outgrowth, was increased in hMSC_topo IIβ(−) (*p* < 0.01) and hMSC_topo IIβ(−)_mN3 cells compared with untransfected cells (*p* < 0.001). On the other hand, a decreased RhoA expression level was detected in hMSC_topo IIβ(+)_mN3 cells (*p* < 0.001), showing a negative correlation between RhoA and topo IIβ gene expressions.

Cdc42, which promotes neurite outgrowth, was downregulated in response to topo IIβ deficiency (*p* < 0.001), whereas the expression level was upregulated in topo IIβ-overexpressing neural cells (*p* < 0.01). Furthermore, topo IIβ overexpression in mN3-treated hMSCs induced an increase in the Cdc42 gene expression level at day 1 (*p* < 0.001) which decreased gradually over the following days.

Except for heterogeneity in the expression levels between days, no significant difference was found in Rac1 expression between each group. In contrast to the known positive regulatory role of Rac1 in neurite outgrowth, RT-qPCR results showed no correlation with topo IIβ deficiency or overexpression.

It has been shown that RhoA activates Rock2, major downstream target protein of RhoA in neural cells, and that the RhoA/Rock2 signaling pathway leads to axon growth inhibition [17]. Depending upon the increased RhoA expression level in topo IIβ-silenced cells, we also investigated the effect of topo IIβ deficiency on Rock2 gene expression. As expected, the Rock2 gene expression level was increased as a result of raised RhoA expression levels in response to topo IIβ silencing. Accordingly, topo IIβ-transfected cells showed a reduction in the expression levels of Rock2 (*p* < 0.01).

Heng et al. demonstrated that topo IIβ was a downstream target of Nurr1, and the expression of the enzyme was downregulated in Nurr1 knockout mice [13]. RT-qPCR analysis was performed to check the expression pattern of Nurr1 in topo IIβ silenced and overexpressing hMSCs. The expression level of Nurr1 was both upregulated in hMSC_topo IIβ(+) and hMSC_topo IIβ(+)_mN3 cells (*p* < 0.001), whereas it was downregulated in hMSC_topo IIβ(−) and hMSC_topo IIβ(−)_mN3 cells (*p* < 0.01) compared with the control during the whole differentiation process. The Nurr1 expression level was highly correlated with topo IIβ in response to topo IIβ silencing and overexpression.

## Discussion

Topo IIβ activity is known to regulate neural differentiation by potentiating neuronal genes that are responsible for neural differentiation processes such as neurite outgrowth [5, 10]. Additionally, preliminary microarray results proposed that topo IIβ silencing appears to be important in several signal transduction pathways which may contribute to neuronal polarization and pathogenesis of neurodegenerative diseases [11]. On the other hand, Rho-GTPases regulate neuronal polarization, and dysregulation of these GTPases lies behind the pathology of neurodegeneration [19]. However, there have been no reports analyzing the link between topo IIβ and Rho-GTPases. In this study, for the first time, we identify that topo IIβ has a regulatory role in the process of axonogenesis via regulation of Rho-GTPases.

Since their neural transdifferentiation capability was first shown, primary hMSCs have been promising candidates for the treatment of damaged neural tissue. However, apart from the apparent ethical issues, the use of primary hMSCs has been limited due to their dependency on conditions such as passage number and donor-related heterogeneity. The low frequency of MSCs necessitates their in-vitro expansion prior to use, and primary cells can be provided for only a limited time before they undergo cellular senescence [31–33]. To obtain sufficient numbers of a homogenous hMSC population and exclude inter-donor variability, the clonally immortalized hMSC line was chosen to make an in-vitro human neural differentiation model in this study. The hMSC line expresses cell surface antigens (CD34^–^, CD45^–^, HLA-DR^–^ CD73^+^, CD90^+^, CD105^+^) commonly used to characterize hMSC populations and can differentiate into mesodermal (osteogenic, adipogenic, chondrogenic) and even nonmesodermal (neural cells) lineages. The hMSC cell line was differentiated into sustainable neuronal morphology by combining several differentiation factors enriched with neurotrophic factors and NGF as well as BDNF [30, 34, 35] and differentiated cells express features specific to mature neural cells, such as Map 2, NF, and Tau. Thus, this immortalized cell line could be an important scientific tool to understand the in-vitro neural differentiation and degeneration mechanisms with a resemblance to the in-vivo situation.

Initially, morphological changes were observed in topo IIβ-silenced cells compared with untransfected cells. Topo IIβ-silenced hMSCs lost their fibroblastic morphology, becoming enlarged and flattened, and continued this morphology at further passages. It is known that topo IIβ expression level and activity decreases during aging [8]. On the other hand, increasing donor age accelerates changes in hMSC morphology, and cells become enlarged in the culture even at early passages [31]. The morphology of hMSCs in the absence of topo IIβ resembles the morphology of cells obtained from aged donors in our previous study [31]. Moreover, silencing of topo IIβ expression before the day of neural induction prevented more than half of the cells from committing to neural differentiation and caused a dramatic decrease in neurite length, confirming previous studies [3, 10, 11].

Attempts were made to understand the mechanism of neural differentiation in the absence of topo IIβ. The most well-established Rho-GTPases (RhoA, Cdc42, and Rac1) regulate axonogenesis in neuronal cells [36]. RhoA has been reported to negatively regulate neurite outgrowth during neural development, and its major downstream effector, Rock2, mediates RhoA-driven neurite retraction [17]. In our study, RhoA and Rock2 gene expressions were upregulated in response to topo IIβ silencing. As Rock2 causes growth cone collapse and neurite retraction, these results suggest that upregulated RhoA and Rock2 gene activity may cause axon growth inhibition in the absence of topo IIβ. Furthermore, it is known that RhoA and Cdc42 can be antagonistic to one another in cells, and a balance in Rho-GTPase activities is required for the regulation of neurite outgrowth [36, 37]. In our study, Cdc42 was shown to be downregulated in topo IIβ-silenced cells, confirming the antagonistic effects of RhoA and Cdc42 in neurite outgrowth. On the other hand, a study by Chen et al. has shown that, during development of mice vertebrate central nervous system (CNS), deletion of Rac1 prevented axonal migration but not axonal outgrowth. In this study, no significant change was observed in the expression of Rac1 in topo IIβ knockdown cells, supporting the hypothesis that Rac1 primarily controls axon guidance rather than outgrowth [38].

Axon growth inhibition, a degenerated neuronal network, and cell death following loss of cellular functions are common characteristics of several neurodegenerative diseases. Involvement of the RhoA/Rock2 signaling pathway has been suggested in neurodegenerative disorders such as AD, PD, HD, and ALS. Some nonsteroidal anti-inflammatory drugs (NSAIDs) have been shown to reduce the risk of neurodegeneration by blocking the RhoA/Rock2 signaling pathway [17]. Inhibition of the RhoA/Rock2 pathway can be a therapeutic lead for diverse neurodegenerative disorders. Despite many treatment strategies for neurodegeneration, no full cure has been achieved because of the limited neurogenesis in the nervous system. Stem cell-based gene therapy shows potential as a powerful means of treating neurodegenerative disorders. Among adult stem cells, MSCs are one of the most extensively studied cell types with respect to their neural differentiation potential, and provide hope of developing therapeutics for neurodegenerative diseases [39]. There is ongoing research for MSCs as a therapeutic tool for AD, PD, and HD [21].

For this reason, we investigated the possible neuroprotective effect of topo IIβ overexpression on the neural differentiated hMSC line to elucidate the further involvement of Rho-GTPases in topo IIβ-mediated axon growth. Topo IIβ overexpression prompted all the cells to exhibit neural cell morphology as characterized by longer neurites. Our results indicate that topo IIβ is necessary for the commitment of the hMSC line to neural differentiation and promoting axon outgrowth.

Previous reports have indicated that RhoA/Rock2 inhibition can induce axonal regeneration after injury [40–44]. Thus, RhoA and Rock2 gene expression levels were analyzed in response to topo IIβ overexpression. Our results demonstrated that overexpression of topo IIβ causes a decrease in RhoA and Rock2 expression. Our findings suggest that topo IIβ induces neurite outgrowth through inhibition of the RhoA/Rock2 pathway. On the other hand, Cdc42 was shown to be upregulated in topo IIβ-overexpressing cells, confirming a leading role in neurite outgrowth [36]. Another possible explanation for this is the opposing functions of RhoA and Cdc42, probably balanced by Rho regulator proteins. In contrast, no significant change was observed in the expression of Rac1, supporting the hypothesis that Rac1 may be responsible for other neurogenesis steps rather than axon growth in the neural differentiation process of hMSCs.

Studies performed in neural cells have found that Rac1 and Cdc42 activities are localized to the tips of the growing neurites [36]. In agreement with these studies, we also detected Rac1 and Cdc42 signals in the growing ends of axons in response to topo IIβ overexpression, confirming the topo IIβ-dependent role of these GTPases in axon elongation. On the other hand, RhoA is localized to the cytoplasm and a low amount of endogenous RhoA is detected in the nuclear fraction [41]. In topo IIβ-overexpressing cells, RhoA signals were enriched in the nucleolus, whereas RhoA was not detectable in the nucleus and was restricted to the cytoplasm in topo IIβ-deficient cells. These data indicate that topo IIβ causes a translocation of RhoA from the cytoplasm to the nucleus. It has been shown that nuclear localization of topo IIβ is highly correlated with its catalytic activity. Active topo IIβ is largely nucleoplasmic, while the nucleolar form is inactive [42]. It is possible that there could be a link between the subcellular localization of RhoA and its activity. In contrast to Dubash et al. [41], who found RhoA can be detected in the nucleus in its GTP-bound active form, our results propose that inactive RhoA could accumulate in the nucleolus where it has limited access to the plasma membrane in topo IIβ overexpressed neural cells. Furthermore, in response to topo IIβ deficiency, RhoA could leave the nucleolus and target the cytoplasmic membrane upon activation.

Our findings suggest that Rho-GTPases can be a downstream target of topo IIβ; however, knowledge on the upstream regulation of topo IIβ is also inadequate. Nurr1, a transcription factor belonging to the steroid/thyroid hormone receptor family, regulates the expression of target genes by binding to DNA. A study with Nurr1 knockout mice revealed that topo IIβ is a downstream protein of Nurr1 and that Nurr1 regulates neurite outgrowth by regulating topo IIβ gene expression [13]. The same study also showed that overexpression of topo IIβ rescues Nurr1 deficiency-induced axon growth inhibition, indicating that topo IIβ is the essential protein responsible for axon growth [13]. In our study, the expression pattern of Nurr1 in topo IIβ-silenced and -overexpressed hMSCs correlated with topo IIβ. One possible explanation for the regulation of Nurr1 gene expression according to topo IIβ in the neural-induced hMSCs line is that the mechanism of topo IIβ expression forms part of a feedback loop to regulate Nurr1 expression.

## Conclusions

Based on the results of this study, it appears that inhibition of the RhoA/Rock2 pathway by topo IIβ overexpression can be used as a strategy to enhance the neural differentiation potential of MSCs and induce neurite outgrowth. A further study exploring the effect of topo IIβ-transfected primary MSC implantation on neurodegenerative disease animal models will provide us with a better understanding regarding the neuroprotector role of topo IIβ in neurodegeneration.

## Additional files

Additional file 1: **Figure S1.** Surface marker expressions of the hMSC line cells by flow cytometry. hMSC line cells were positive for CD90 (99.7%), CD105 (99.7%), and CD73 (99.8%), and negative for CD45 (0.1%), CD34 (0.5%), and HLA-DR (0.1%). (TIFF 553 kb)

Additional file 2: **Figure S2.** Multilineage mesodermal differentiation of the hMSC line cells. Undifferentiated (control) hMSCs (A), adipogenic differentiation stained with Oil Red O (B), osteogenic differentiation stained with Toluidine Blue (C), and chondrogenic differentiation stained with Alcian Blue solution (D). Images ×10. (TIFF 6680 kb)

## Abbreviations

AD: Alzheimer’s disease
ALS: Amyotrophic lateral sclerosis
BM-hMSC: Bone marrow-derived human mesenchymal stem cell
CGN: Cerebellar granule neuron
CN: Cortical neuron
dbCAMP: Dibutyryl cyclic AMP
DMEM-LG: Dulbecco’s modified Eagle’s medium low glucose
DRG: Dorsal root ganglion
EGFP: Enhanced green fluorescent protein
FBS: Fetal bovine serum
FGF: Fibroblast growth factor
HD: Huntington’s disease
hEGF: Human epidermal growth factor
hMSC: Human mesenchymal stem cell
IBMX: 3-Isobutyl-1-methylxanthine
MAP2: Microtubule-associated protein
mN3: Modified N3 medium
NF: Neurofilament
NGF: Nerve growth factor
NGS: Normal goat serum
NHS: Normal horse serum
NSAID: Nonsteroidal anti-inflammatory drug
p: Passage
PBS: Phosphate-buffered saline
PD: Parkinson’s disease
rhBDNF: Recombinant human brain-derived neurotrophic factor
rhFGF: Recombinant human fibroblast growth factor
RT-qPCR: Reverse transcription quantitative polymerase chain reaction
SD: Standard deviation
topo II: Topoisomerase II
