# Supplementary figures and images for: DNA topoisomerase IIβ stimulates neurite outgrowth in neural differentiated human mesenchymal stem cells through regulation of Rho-GTPases (RhoA/Rock2 pathway) and Nurr1 expression
Source: Stem Cell Res Ther. 2018 Apr 25;9:114. doi: 10.1186/s13287-018-0859-4 (PMC5918966; doi:10.1186/s13287-018-0859-4)

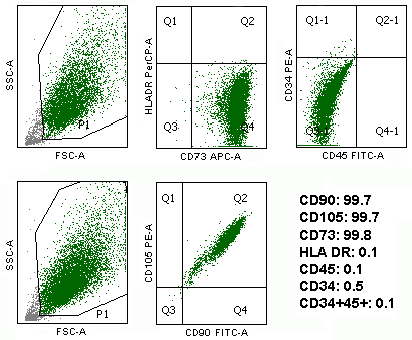

Supplement: Supplementary file 1 — Figure S1. Surface marker expressions of the hMSC line cells by flow cytometry. hMSC line cells were positive for CD90 (99.7%), CD105 (99.7%), and CD73 (99.8%), and negative for CD45 (0.1%), CD34 (0.5%), and HLA-DR (0.1%). (TIFF 553 kb) [file 13287_2018_859_MOESM1_ESM.tif]

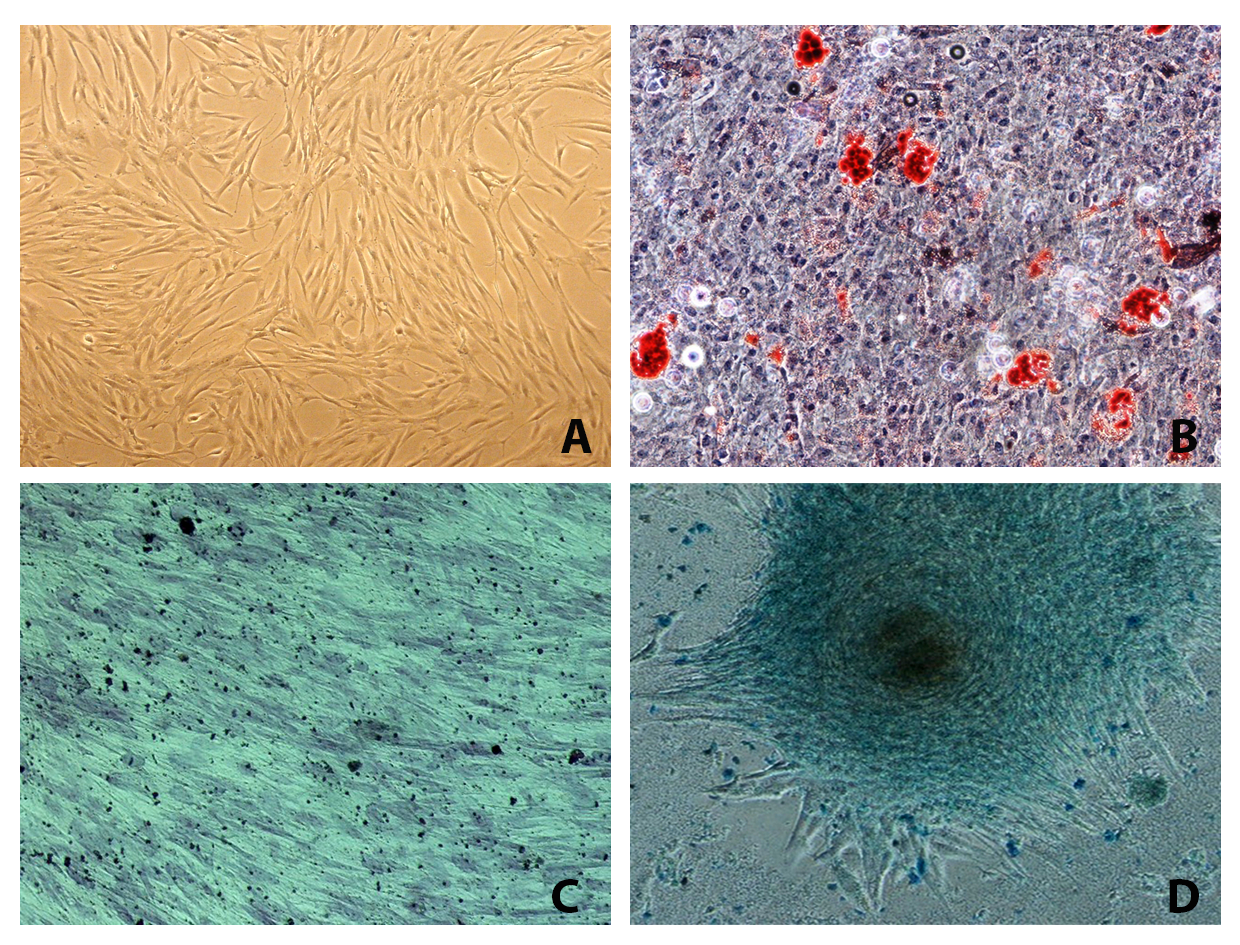

Supplement: Supplementary file 2 — Figure S2. Multilineage mesodermal differentiation of the hMSC line cells. Undifferentiated (control) hMSCs (A), adipogenic differentiation stained with Oil Red O (B), osteogenic differentiation stained with Toluidine Blue (C), and chondrogenic differentiation stained with Alcian Blue solution (D). Images ×10. (TIFF 6680 kb) [file 13287_2018_859_MOESM2_ESM.tif]
